# Animal ICU… Why not also use the existing veterinary ICUs?

**DOI:** 10.1186/s13613-019-0568-x

**Published:** 2019-08-16

**Authors:** Florent Baudin, Céline Pouzot-Nevoret, Vanessa Louzier, Isabelle Goy-Thollot, Anthony Barthélemy, Stéphane Junot, Jeanne-Marie Bonnet-Garin, Bernard Allaouchiche

**Affiliations:** 1 Service de réanimation pédiatrique, Hôpital Femme Mère Enfant, Hospices Civils de Lyon, 59 Bd Pinel, 69500 Bron, France; 2 Agressions Pulmonaires et Circulatoires dans le Sepsis (APCSe), VetAgro Sup, UPSP 2016.A101, Université de Lyon, 69280 Marcy l’étoile, France; 3 Intensive Care Unit (SIAMU), VetAgro Sup, Université de Lyon, 69280 Marcy l’Etoile, France; 4 Département d’anesthésie et de reanimation, Hospices Civils de Lyon, Lyon Sud Teaching Hospital, 69495 Pierre-Bénite, France

To the editor,

We have read with interest the article published recently in the journal by Guillon et al. “Preclinical septic shock research: why we need an animal ICU” [1]. The authors review the limitations of current animal models in septic shock especially the lack of heterogeneity and comorbidities, and argue the need to develop animal intensive care unit (ICU) to better reflect the reality of human management.

We agree with the authors that rodents poorly reflect human conditions, and that financial and ethical issues are the main limitations to perform experiments in larger species. However, another way to explore therapies in sepsis, which was not mentioned by the authors, is the use of clinical models (in particular canine and feline) treated in existing veterinary ICUs. Our young research unit “*Agressions Pulmonaires et Circulatoires dans le Sepsis*” (APCSe) is composed of veterinary and medical physicians, and we have a platform for ovine and porcine experiments where we are able to reproduce ICU conditions for large-animal preclinical models [2, 3]. We also have access to a veterinary ICU at the veterinary school in Lyon (VetAgro Sup) where dogs and cats are treated for severe sepsis. For example, we previously explored hemodiafiltration [4] or hemostatic disorders in dogs suffering from multiple organ failure due to leptospirosis [5]. This approach provides solutions to the limitations highlighted by the authors: different weights, different ages and not only two conditions (alive or dead), but also animals having received organ support for several days (Fig. 1).

**Fig. 1:**
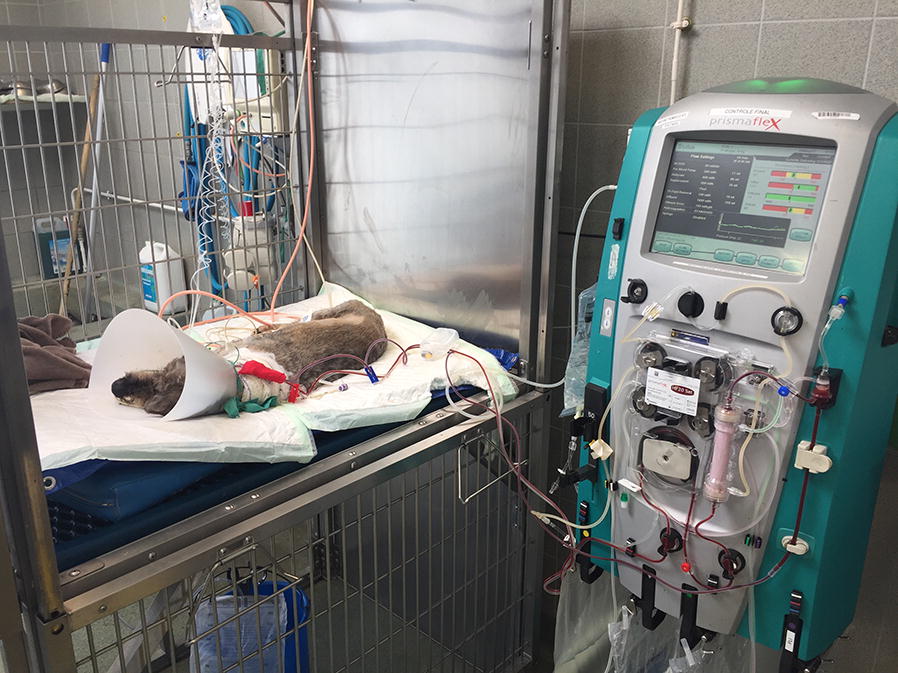
Example of a dog treated for multiple organ failure secondary to leptospirosis infection in the veterinary intensive care unit

Another point is that this partnership allows not only to do translational research from animal to human, but also to implement methods used in human medicine to the veterinary ICU; for instance, recently, we introduced the use of high-flow nasal cannula in the veterinary ICU to treat respiratory failure due to viral or bacterial infections in dogs and cats (Fig. 2).

**Fig. 2:**
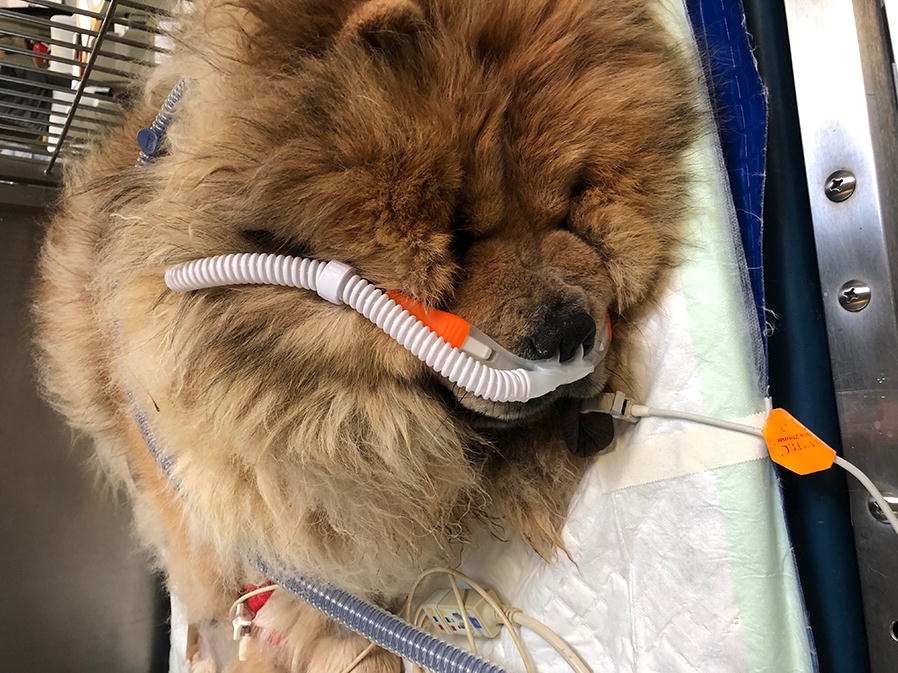
Example of a dog treated with high-flow nasal cannula because of septic respiratory failure

In conclusion, animal ICU is probably the next step for research but we believe also that the future is the pooling of veterinary and medical expertise to perform research that will benefit both humans and animals in the ICU which is the “one health” concept.

## Data Availability

Not applicable.

## Abbreviation

ICU: intensive care unit
